# The Passage of Blood by the Bowel

**Published:** 1905-01-07

**Authors:** 


### THE PASSAGE OF BLOOD BY THE BOWEL.

In a clinical lecture which he gave recently at Guy's Hospital, and which is reported in the *Guy's Hospital Gazette*, Dr. Hale White illustrated by a number of cases the value as a symptom of blood passing by the rectum, and incidentally he called attention to the fact that the symptom is not inquired for by the medical attendant nearly as often as it should be. Blood in the stools is not difficult to recognise; even if it comes from high up in the bowel and is so altered that its presence cannot be ascertained with certainty by mere inspection all that is necessary is to apply a little tincture of guaiacum and ozonic ether and if a blue colour is developed it shows that blood is present. One of the most frequent causes of loss of blood by the bowel is the presence of hæmorrhoids, and the daily repetition of the loss may lead to severe anæmia and a marked degree of ill-health. It is no infrequent thing to come across a woman who is pale and generally out of health, while the first inquiry and examinasion fail to reveal the cause of her ill-health. She is simply unsatisfactory and never feels quite up to the mark. Such a patient may wander from doctor to doctor without getting any relief, because they fail to realise that all the woman's troubles may be produced by hæmorrhoids. But when the true nature of the malady is recognised, and the hæmorrhoids are medically treated or removed by operation, it is a striking thing to see the improvement in health which follows. In illustration of these facts Dr. Hale White mentioned the case of a woman who passed large quantities of blood by the bowel, and who lost so much weight and showed such constitutional disturbance that there seemed no reasonable doubt that she was suffering from malignant disease of the bowel. A laparotomy was performed but no growth was found. Events proved that all her symptoms had been due to hæmorrhoids and a Whitehead's operation brought complete relief. Duodenal ulcer is another condition which may cause severe anæmia through intestinal hæmorrhage which is very likely to be overlooked by the medical attendant, and the more so as the blood comes from high up in the bowel and so is usually much altered in appearance, often requiring the tincture of guaiacum of ozonic ether test before it can be recognised with certainty. But this is not always so, and because in any particular case bright red blood is passed, there are no proper grounds for assuming that it is impossible for the bleeding to come from high up in the bowel. Indeed, Dr. Hale White has known instances of hæmorrhage undoubtedly from a duodenal ulcer in which the blood passed has been bright red, although he does not believe it is ever bright red if it comes from the stomach. The necessity for examining the fæces for blood in obscure cases of anæmia is well shown in duodenal ulcer, for here the bleeding may be the only diagnostic symptom, and if it is overlooked the proper line of treatment will not be chosen, and the patient's life may be lost in consequence. Pneumonia is not generally recognised as a possible cause of hæmorrhage by the bowel, but, although rare, intestinal hæmorrhage does sometimes occur in the course of acute pneumonia, and the possibility should be recognised. Henoch's purpura and Bright's disease, too, are conditions which may be overlooked as causes of bleeding from the intestines. Other conditions to which Dr. Hale White referred are malignant disease, presence of a foreign body, malingering, swallowing of blood after hæmoptysis or other cause, intussusception, enteric fever, gastroenteritis, constipation, tuberculosis, purpura hæmorrhagica, colitis, dysentery, ankylostomiasis, and thrombosis of the mesenteric vessels.

